# Targeting of Protein Kinase CK2 Elicits Antiviral Activity on Bovine Coronavirus Infection

**DOI:** 10.3390/v14030552

**Published:** 2022-03-07

**Authors:** Ailyn C. Ramón, George V. Pérez, Evelin Caballero, Mauro Rosales, Daylén Aguilar, Dania Vázquez-Blomquist, Yassel Ramos, Arielis Rodríguez-Ulloa, Viviana Falcón, María Pilar Rodríguez-Moltó, Ke Yang, Yasser Perera, Silvio E. Perea

**Affiliations:** 1 Molecular Oncology Group, Department of Pharmaceuticals, Biomedical Research Division, Center for Genetic Engineering and Biotechnology, Havana 10600, Cuba; ailyn.ramon@cigb.edu.cu (A.C.R.); george.perez@cigb.edu.cu (G.V.P.); evelin.caballero@cigb.edu.cu (E.C.); mauro.rosales@fbio.uh.cu (M.R.); daylen.aguilar@cigb.edu.cu (D.A.); 2 Department of Animal and Human Biology, Faculty of Biology, University of Havana, Havana 10400, Cuba; 3 Pharmacogenomic Group, Department of System Biology, Biomedical Research Division, Center for Genetic Engineering and Biotechnology, Havana 10600, Cuba; dania.vazquez@cigb.edu.cu; 4 Mass Spectrometry Laboratory, Proteomics Group, Department of Systems Biology, Biomedical Research Division, Center for Genetic Engineering and Biotechnology, Havana 10600, Cuba; yassel.ramos@cigb.edu.cu (Y.R.); arielis.rodriguez@cigb.edu.cu (A.R.-U.); 5 Microscopy Laboratory, Department of System Biology, Biomedical Research Division, Center for Genetic Engineering and Biotechnology, Havana 10600, Cuba; viviana.falcon@cigb.edu.cu; 6 Department of Agricultural Research, Animal Biotechnology Division, Center for Genetic Engineering and Biotechnology, Havana 10600, Cuba; pilar.rodriguez@cigb.edu.cu; 7 China-Cuba Biotechnology Joint Innovation Center, Yongzhou Zhong Gu Biotechnology, Yongzhou 425000, China

**Keywords:** bovine coronavirus, protein kinase CK2, kinase inhibitor, CIGB-325

## Abstract

Coronaviruses constitute a global threat to the human population; therefore, effective pan-coronavirus antiviral drugs are required to tackle future re-emerging virus outbreaks. Protein kinase CK2 has been suggested as a promising therapeutic target in COVID-19 owing to the in vitro antiviral activity observed after both pharmacologic and genetic inhibition of the enzyme. Here, we explored the putative antiviral effect of the anti-CK2 peptide CIGB-325 on bovine coronavirus (BCoV) infection using different in vitro viral infected cell-based assays. The impact of the peptide on viral mRNA and protein levels was determined by qRT-PCR and Western blot, respectively. Finally, pull-down experiments followed by Western blot and/or mass spectrometry analysis were performed to identify CIGB-325-interacting proteins. We found that CIGB-325 inhibited both the cytopathic effect and the number of plaque-forming units. Accordingly, intracellular viral protein levels were clearly reduced after treatment of BCoV-infected cells, with CIGB-325 determined by immunocytochemistry. Pull-down assay data revealed the physical interaction of CIGB-325 with viral nucleocapsid (N) protein and a group of bona fide CK2 cellular substrates. Our findings evidence in vitro antiviral activity of CIGB-325 against bovine coronavirus as well as some molecular clues that might support such effect. Altogether, data provided here strengthen the rationale of inhibiting CK2 to treat betacoronavirus infections.

## 1. Introduction

Coronaviruses (CoVs) comprise a diverse group of enveloped positive-strand RNA viruses in the family *Coronaviridae* that infect a wide variety of hosts, including mammals and birds, and cause several respiratory and enteric diseases [1,2]. These viruses include infectious bronchitis virus (IBV) in chickens, porcine epidemic diarrhea virus (PEDV), transmissible gastroenteritis virus (TGEV), swine acute diarrhea syndrome-CoV (SADS-CoV), bovine coronavirus (BCoV), and many others [3]. Human coronaviruses HCoV-229E, HCoV-OC43, HCoV-HKU1, HCoV-NL63 are continuously circulating in the human population, causing the common cold. However, three highly pathogenic coronaviruses (SARS-CoV, MERS-CoV, and SARS-CoV-2) that belong to betacoronavirus (β-CoV) genus have crossed species to cause severe human respiratory disease [3]. In 2002, a human pathogenic CoV, severe acute respiratory syndrome (SARS-CoV), caused more than 8000 human infections in 2002–2003, with a case fatality about 10% [4]. A decade later, a novel lethal zoonotic disease appeared in the Arabian Peninsula, caused by the Middle East respiratory syndrome coronavirus (MERS-CoV), with a 36.1% mortality rate according to the World Health Organization (WHO) [5]. Finally, a novel coronavirus SARS-CoV-2 emerged from Wuhan, China, in December 2019 and three months later became a pandemic disease with more than 350 million people infected and 5 million deaths across the world (www.worldometers.info/coronavirus, accessed on 31 January 2022).

In this context, vaccination represents a reasonable strategy for preventing or avoiding coronavirus infections; however, vaccines fail, in part, due to virus resistance emergence [6]. The frequency and extension of the abovementioned epidemiological events highlight the urgent necessity of therapeutic solutions for treating viral infections. Taking into consideration that SARS-CoV-2 outbreak occurred in a scenario with no established active molecules against betacoronavirus, the availability of effective pan-coronavirus antiviral drugs is required to control re-emerging virus outbreaks. Considering the urgent need of drug development, the re-purposing of established drugs may provide a straightforward road for identifying molecules with potential use in the clinical practice against β-CoV infections [7,8].

Traditional antiviral strategies directed to viral targets often yield drug resistance; therefore, targeting relevant host factors involved in viral replication guarantee therapeutics with a wide-spectrum activity since families of viruses share common cellular signaling pathways and processes [9,10]. Within host factors that viruses hijack during the infection process, kinases proteins play a fundamental role by phosphorylating viral and host substrates [11]. Recently, protein kinase CK2 has emerged as a relevant therapeutic target in the treatment of β-CoV infections, supported by its genetic and pharmacologic inhibition [12,13]. Remarkably, during SARS-CoV-2 infection CK2 interacts with the N protein, and this protein kinase was found dramatically upregulated in infected cells. The foregoing was reinforced by an increased phosphorylation of well-characterized substrates involved in the cytoskeleton organization, mainly on the filopodia protrusions that promote virus egress and rapid cell-to-cell spread [10].

CIGB-325 (formerly CIGB-300) is a synthetic peptide designed to target a subset of CK2 substrates through binding to the conserved phosphoacceptor sites, and recently it has shown a direct impact over the CK2 enzymatic activity as classical inhibitors [14,15,16]. This biochemical feature supports the first-in-class attribute of CIGB-325 and entails important pharmacological differences compared with other available CK2 inhibitors. Clinical data have confirmed that CIGB-325 peptide is safe and well-tolerated when administered by intravenous infusion in cancer patients [17,18]. Preliminary evidence of CIGB-325 antiviral activity has been shown in two different settings. For instance, in vitro anti-HIV activity was reported, as the peptide interferes with a putative B23/NPM1-Rev interaction in cells and subsequently downregulates Rev-dependent gene expression [19]. Furthermore, a phase I/II clinical trial with CIGB-325 showed benefits in COVID-19 patients with pneumonia, where significant reduction in the pulmonary lesions and quick improvement in the clinical status at day 7 was observed [20].

Some molecular and pathophysiological features of the disease caused by human CoV can be recapitulated in animal CoV diseases, where animal coronavirus models are suitable and alternative systems exist for testing pan-coronavirus therapies [21]. Such models have the additional advantage of not requiring restrictive BSL-3 facilities. In this work we aimed to explore the putative antiviral activity of the anti-CK2 inhibitor CIGB-325 against β-CoV infections using a bovine coronavirus (BCoV) model. To note, this virus belongs to the β-CoV genus that includes murine hepatitis coronaviruses (MHV), porcine hemagglutinating encephalomyelitis virus (HEV), rat coronavirus (RtCoV), human respiratory coronavirus HCoV-OC43, and severe acute respiratory syndrome (SARS-CoV, SARS-CoV-2) [22]. BCoVs cause respiratory and enteric diseases in cattle and other ruminants, and they are zoonotically transmissible among species since BCoV-like viruses have been detected in wild ruminants and humans [23]. During the disease, the virus infects both the small and large intestines, destroying villi and leading to different clinical syndromes in cattle: winter dysentery characterized by hemorrhagic diarrhea in adults and neonatal calf diarrhea and respiratory infections in cattle of different ages [24]. BCoV shares biological pathogenic and pneumoenteric properties with species of coronavirus related to SARS-CoVs [25]. Even though BCoV uses a different cellular receptor to enter into the cells, replication, assembly, and final egress of viral particles may share common viral and cellular factors [26,27].

In this work, we tested for the first time the in vitro antiviral activity of the anti-CK2 peptide CIGB-325 against BCoV infection, a model for β-CoV. We provide experimental evidence in BCoV-infected MDBK cells that contribute to the characterization of the antiviral mechanism of this peptide.

## 2. Materials and Methods

### 2.1. Cell Culture and BCoV Virus

Madin-Darby Bovine Kidney cell line (MDBK) (ATCC^®^ CCL-22™) was maintained in Dulbecco’s modified Eagle’s medium (DMEM) (Gibco, Waltham, MA, USA), supplemented with 10% inactivated fetal bovine serum (FBS) (Invitrogen, Carlsbad CA, USA) at 37 °C and 5% CO_2_. The BCoV (strain Mebus) was obtained from the Pasteur Institute in São Paulo, Brazil, and titrated in serial 1 log dilutions (from 1 log to 11 log) to obtain a 50% tissue culture infective dose (TCID_50_) on 96-well culture plates of MDBK cells. The plates were observed daily during 4 days for the presence of cytopathic effect (CPE) using an inverted optical microscope. The viral titer was calculated according to the Reed–Muench method based on eight replicates for titration.

### 2.2. Compounds

CIGB-325 and F20-2 peptides (Center for Genetic Engineering and Biotechnology, Havana, Cuba) were dissolved as a 10 mM stock in PBS at room temperature for 5 min. For each experiment, a freshly-made stock was used. Interferon alpha-2b (Center for Genetic Engineering and Biotechnology, Havana, Cuba) was dissolved in distilled water to 10^6^ IU/mL, and CX-4945 was obtained from SelleckChem (Munich, Germany) and was resuspended to a stock solution of 10 mM in dimethyl sulfoxide (DMSO). The drugs were diluted directly into growth media just prior to use.

### 2.3. Cell Cytotoxicity Assay and Drug Treatments

Cell cytotoxicity was determined using crystal violet staining. Briefly, 60,000 MDBK cells were seeded in flat-bottom 96-well plates per well in DMEM medium with 10% fetal bovine serum (FBS), and a curve of serial dilutions (1:2) of CIGB-325 (3.12–200 μM) was added in triplicate. After 4 days, crystal violet stain was conducted, as described in 2.4. The half-cytotoxic concentration CC_50_ was estimated from the fitted dose–response curves using CalcuSyn software (Biosoft, Cambridge, UK).

### 2.4. Cytopathic Effect Assay

To evaluate the antiviral effect against BCoV, 260,000 MDBK cells were seeded in 24-well cell culture plates per well and incubated overnight at 37 °C in 5% CO_2_. After incubation, selected concentrations of CIGB-325, CX-4945, or F20-2 negative control peptide were added to cell monolayers for 1 h in serum-free DMEM. Interferon alpha-2b (IFN alpha-2b) at 500 IU/ mL was employed in this study as a positive control. Subsequently, 14,000 TCID_50_ of virus in 200 µL was added to each well of the plate (MOI = 0.01). After 1 h of incubation, the final volume was completed up to 1 mL, and the appropriate drug concentration was maintained. Incubation was prolonged for 4 days at 37 °C in 5% CO_2_, and the CPE was finally revealed by crystal violet staining. The antiviral activity rate was expressed as the drug concentration that protects 50% of CPE. The half-inhibitory concentration (IC_50_) values were estimated from the fitted dose–response curves using CalcuSyn software (Biosoft, Cambridge, UK).

### 2.5. Crystal Violet Assay

Crystal violet staining was used to evaluate cytotoxicity of CIGB-325 and verify the viral cytopathic effect perceived by visual observation. After the incubation period, medium was removed from each well and crystal violet (1%) (Sigma, St. Louis, MO, USA) was added and incubated for 5 min at room temperature. After dye removal, wells were washed with water. Finally, absorbance at 578 nm was read using a CLARIOstar^®^ high-performance monochromator multimode microplate reader (BMG LABTECH, Ortenberg, Germany). The percentage cell viability was calculated using the following formula: Cell viability (%) = ((OD of treated cells)/(OD of cell control)) × 100.

### 2.6. Plaque Reduction Assay

Viral titers in supernatants of drug-untreated and treated cells were evaluated using a plaque reduction assay. Briefly, MDBK cells were seeded at 260,000 cells per well in 24-well cell culture plates and incubated overnight at 37 °C in 5% CO_2_. Subsequently, 200 μL of supernatants in 10-fold serial dilution was added to the MDBK monolayers. After 1 h of incubation at 37 °C and 5% CO_2_, the viral inoculum was aspirated, and 0.5 mL of carboxymethylcellulose (Sigma, St. Louis, MO, USA) overlay with DMEM and supplemented with 2% FBS was added to each well. After 4 days of incubation, the cells were fixed and stained with Naphthol Blue Black (0.1%) (Sigma, St. Louis, MO, USA). Finally, plaques were counted visually, and the virus titer as plaque-forming units (PFU) per mL was calculated.

### 2.7. Quantitative Real-Time PCR Assays

MDBK cells were plated on 24-well cell culture plates at 260,000 cells per well and incubated overnight at 37 °C and 5% CO_2_. Once cell monolayers were established, CIGB-325 (15 µM, 30 μM), F20.2 (30 μM), CX-4945 (1.25 μM), or vehicle (PBS) was added for 1 h in serum-free DMEM. Subsequently, 14,000 TCID_50_ of virus in 200 µL was added to each well (MOI = 0.01). After 1 h of incubation, final volume was completed up to 1 mL, and the appropriate drug’s concentration was maintained for 24 h. Three replicates per condition were used. After incubation time, the culture medium was withdrawn, and the cells were washed with PBS and suspended in 350 µL of Lysis Buffer (with 1% of β-mercaptoethanol, Sigma, St. Louis, MO, USA) for RNA isolation (AllPrep DNA/RNA/miRNA Universal Kit, Qiagen, Valencia, CA, USA), according to the manufacturer protocol. All RNA samples were checked by Nanodrop spectrophotometer to measure concentration (ng/µL) and OD relation (260/280 nm). Quality control parameters were fulfilled by all the samples (100% OD 260/280 nm between 1.7 and 2.2). Complementary (c)DNAs were obtained from 500 ng of total RNAs, using the Transcriptor First Strand cDNA Synthesis Kit package (Roche, Mannheim, Germany), following manufacturer instructions. The qPCR reactions were set up in 20 µL using LightCycler^®^ 480 SYBR Green I Master 2x (Roche, Mannheim, Germany), 300 nM of oligonucleotides, and 1:10 dilutions of each cDNA, with three replicates per sample. We amplified two genes for normalization *GAPDH* (glyceraldehyde-3-phosphate dehydrogenase) and *HMBS* (hydroxymethyl-bilane synthase) and the transcript encoding for N protein from BCoV. In parallel, we used a plasmid with the BCoV N protein transcript cloned so that it was amplified from serial dilutions as a standard curve. This standard curve was used for N copy number calculations. All the oligonucleotides were synthetized in the Synthesis Department at CIGB (Table S1). Runs were carried out in the LightCycler^®^480II equipment (Roche, Mannheim, Germany) in a 96-well format and SYBR Green Probe II mode with a standard program with 45 cycles. Fold changes of N transcript expression with each treatment were calculated concerning the Virus control using REST 2009 program (v2.0.13, Qiagen GbmH, Munich, Germany) [28], after normalization with *GAPDH* and *HMBS* genes, using Ct values and reaction efficiencies per amplicon calculated in LinReg 2009 (v 11.3) [29]. Statistical differences are reported in this program, with a *p* value-associated significance of *p* < 0.05 [28]. Additionally, we report a copy number of N transcripts by extrapolation of Ct values into the regression formula from the standard curve (N-BCoV encoding plasmid copy number vs Ct).

### 2.8. BCoV Viral Proteins Detection by Western Blot

MDBK cells were plated on 24-well cell culture plates at 260,000 cells per well and incubated overnight at 37 °C in 5 % CO_2_. Once cell monolayers were established, CIGB-325 (30 μM) or vehicle (PBS) was added for 1 h in serum-free DMEM. Subsequently, 14,000 TCID_50_ of virus in 200 µL was added to each well of the plate (MOI = 0.01). After 1 h of incubation, final volume was completed up to 1 mL, and the appropriate drug’s concentration was maintained for 24 h. After incubation, the culture medium was withdrawn, and the cells were washed with PBS and lysed in RIPA buffer containing protease/phosphatase inhibitor (Thermo Fisher Scientific, Waltham, MA, USA). Equal amounts of protein (30 µg/sample) were resolved in 12% SDS-PAGE. Proteins were then transferred to nitrocellulose membrane and immunoblotted with 30 µg/mL of a human polyclonal IgG antibody against whole SARS-CoV-2 for 2 h at room temperature. This antibody was generated by the Center for Genetic Engineering and Biotechnology (Havana, Cuba) from a COVID-19 convalescent patient with higher titers and validated by ELISA and Western blot. Detection was performed with peroxidase-conjugated anti-human IgG 1:100 (Jackson ImmunoResearch, West Grove, PA, USA), and signal was developed using SuperSignal West Pico Chemiluminescent Substrate (Thermo Fisher Scientific, Waltham, MA, USA).

### 2.9. BCoV Viral Proteins Detection by Immunocytochemistry

MDBK cells were plated on eight-well glass slides and incubated overnight at 37 °C and 5% CO_2_. After incubation, cells were pre-treated for 1 h with CIGB-325 (30 μM) or vehicle (PBS) and infected with 100 µL of virus at a concentration of 70,000 TCID_50_/well (MOI = 0.1). After 1 h of incubation, final volume was completed up to 500 µL, and the appropriate drug’s concentration was maintained for 16 h and 24 h. Subsequently, the cells were washed with cold PBS three times and fixed in 4% formaldehyde for 10 min at 4 °C. After permeabilization with 0.5% Triton X-100 for 10 min, cells were blocked by incubation with 4% bovine serum albumin (Sigma, St Louis, MO, USA) in PBS for 30 min at room temperature, washed again, and incubated with 30 μg/mL human polyclonal anti- SARS-CoV-2 for 2 h at room temperature. Then, peroxidase-conjugated anti-rabbit secondary antibody 1:100 (Sigma, St. Louis, MO, USA) was incubated for 1 h at room temperature and washed 3 times with PBS. Finally, AEC substrate (Abcam, Cambridge, UK) was added and coverglass was mounted using 40% glycerol mounting medium and analyzed using a BX43 upright microscope (Olympus America Inc., Waltham, MA, USA).

### 2.10. Pull-Down Assay

CIGB-325-interacting proteins were identified by in vitro pull-down followed by tandem mass spectrometric analysis (LC-MS/MS). MDBK cells were seeded at 3,600,000 cells in 60 mm dishes and infected with 500 µL of virus at 70,000 TCID_50_/dish (MOI = 0.01). After 48 h post-infection, cells were collected, washed, and lysed in hypotonic PBS solution (1×) (Sigma, St. Louis, MO, USA) containing 1 mM DTT (Sigma, St. Louis, MO, USA), TritonX-100 (1%), and complete protease inhibitor (Roche, Basel, Switzerland). Cellular lysates were cleared by centrifugation, and 300 µg of total protein was incubated with biotin-tagged CIGB-325 (100 μM) or medium alone for 30 min at room temperature and added to 30 µL of pre-equilibrated streptavidin–sepharose matrix (GE Healthcare, Chicago, IL, USA). Following 1 h at 4 °C, the matrix was collected by centrifugation and extensively washed with cold PBS 1 mM with DTT. CIGB-325-interacting proteins were eluted, resolved in an SDS-PAGE gel, and processed as described later. For in vivo pull-down assays, BCoV-Mebus-infected and uninfected MDBK cells were treated with biotin-tagged CIGB-325 (100 μM) or medium alone for 30 min at 37 °C in 5% CO_2_. Subsequently, cells were collected and pull-down assay was conducted as mentioned above. Proteins bound to streptavidin–sepharose were eluted, resolved in a 12 % SDS-PAGE gel, and transferred onto nitrocellulose membranes. For Western blot analysis, a human polyclonal IgG anti-SARS-CoV-2 and a mouse monoclonal antibody against CK2α (Abcam, Cambridge, UK) were used as primary antibodies. Detection was performed with peroxidase-conjugated anti-mouse IgG 1:5000 (Sigma, St. Louis, MO, USA) and anti-human IgG 1:100 (Jackson ImmunoResearch, West Grove, PA, USA). In parallel, untreated cells were subjected to the same experimental procedure to identify those proteins non-specifically bound to streptavidin–sepharose matrix.

### 2.11. LC-MS/MS Analysis and Protein Identification

Proteins bound to streptavidin–sepharose matrix of both CIGB325 treated sample and background control were processed by the FASP method using Microcon 30 k centrifugal ultrafiltration units (Merck, Darmstadt, Germany) [30]. A volume corresponding to 4 µg of the peptide mixture was separated by a Proxeon Easy-nLC System (Thermo Fisher Scientific, Waltham, MA, USA) using an RP-C18-A2 column (3 μm), 75 μm i.d. × 10 mm (Thermo Fisher Scientific, Waltham, MA, USA), connected online to a hybrid quadrupole orthogonal acceleration tandem mass spectrometer QTof-2 (Waters Corp., Milford, MA, USA). The Masslynx system (version 3.5) from Waters (Milford, MA, USA) was used for data acquisition and processing. Protein identification based on MS/MS spectra was made against the Swiss-Prot database by using the Mascot database search engine (version 2.5). False discovery rate (FDR) was set to 1% for peptide and protein identification. Proteins identified uniquely in the CIGB-325-treated sample were considered as CIGB325-interacting proteins. Protein–protein interaction (PPI) networks were generated using information retrieved from the STRING database [31], and protein kinase CK2 substrates among CIGB-325-interacting proteins were identified based on the Meggio and Pinna dataset [32], the list of high confidence CK2 substrates reported by Bian et al. [33], and the PhosphoSitePlus database (www.phosphosite.org, accessed on 6 June 2021).

### 2.12. Confocal Microscopy

MDBK cells were plated on 8-well glass slides and incubated overnight at 37 °C and 5% CO_2_. After incubation, cells were infected with 100 µL of virus at a concentration of 70,000 TCID_50_/well (MOI = 0.1). Uninfected cells were established as a negative control of the experiment. After 48 h post-infection, cells were treated with fluorescein-tagged CIGB-325 (CIGB-325-F) (30 μM) or vehicle (PBS) for 30 min at 37 °C and 5% CO_2_. Subsequently, cells were washed with cold PBS three times and fixed in 10% formalin for 10 min at 4 °C. After permeabilization with 0.2% Triton X-100 for 10 min, cells were blocked by incubation with 4% bovine serum albumin (Sigma, St. Louis, MO, USA) in PBS for 30 min at room temperature and then washed. BCoV N protein was detected by employing 30 μg/mL of a rabbit polyclonal anti-SARS-CoV-2 N protein specific for the M20P19 peptide (N protein linear epitope) for 2 h at room temperature. The antibody was generated by Center for Genetic Engineering and Biotechnology (Havana, Cuba) and validated by ELISA, Western blot, and immunohistochemistry [34]. Finally, anti-rabbit IgG Alexa Fluor 594 conjugate at 1:250 dilution (Cell Signaling Technology, Danvers, MA, USA) was incubated for 1 h at room temperature and washed 3 times with PBS. Coverglasses were mounted using Vectashield mounting medium with DAPI (Vector Laboratories, Burlingame, CA, USA) and analyzed using an Olympus FV1000 confocal laser scanning microscopeIX81 laser scanning fluorescence microscope (Olympus, Tokyo, Japan). Images were acquired with UPLSAPO 40× immersion objective and processed using Olympus FluoView software (v4.0) (Olympus, Tokyo, Japan). Five optical fields or Z-stacks were examined for each experimental condition.

### 2.13. CK2 Signaling Experiments

MDBK cells were plated on 24-well cell culture plates at 260,000 cells per well and incubated overnight at 37 °C and 5% CO_2_. Once cell monolayers were established, 14,000 TCID_50_ of virus in 200 µL was added to each well of the plate (MOI = 0.01). After 1 h of incubation, final volume was completed up to 1 mL of serum-free DMEM and incubated. After 24 h post-infection, cells were treated with CIGB-325 (30 μM) or vehicle (PBS) in serum-free DMEM for 45 min at 37 °C in 5% CO_2_. Subsequently, the culture medium was withdrawn and the cells were washed with PBS, and Western blot was carried out as described (2.7). Primary antibodies against phospho-RPS6 (S235/236) and total RPS6 (Cell Signaling Technology, Danvers, MA, USA) were used, and detection was performed with peroxidase-conjugated anti-rabbit IgG 1:5000 (Sigma, St. Louis, MO, USA).

### 2.14. Statistical Analysis

Differences between groups were determined using one-way ANOVA followed by Dunnett’s multiple comparisons test. Analyses were performed in GraphPad Prism (v6.01) software for Windows (GraphPad Software, Inc., San Diego, CA, USA). All experiments were conducted at least in triplicates and differences were considered significant for a *p*-value < 0.05.

## 3. Results

### 3.1. CIGB-325 Exhibits Antiviral Effect on Bovine Coronavirus Infected Cells

**Figure 1:**
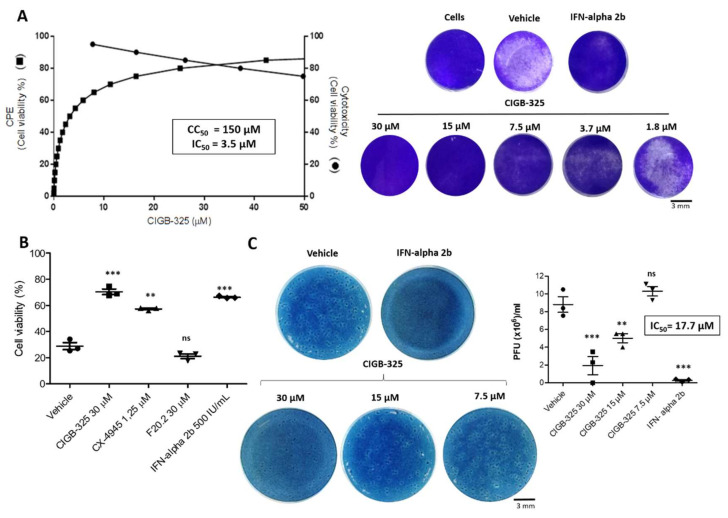
In vitro antiviral activity of CIGB-325 against BCoV-Mebus. MDBK cells were pre-treated with CIGB-325 at the indicated doses for 1 h, and virus (14,000 TCID_50_/well) was then added to allow attachment for 1 h. Subsequently, the cells were incubated in the presence of the indicated drug concentration for 4 days. (**A**) IC_50_ (left axis) and CC_50_ (right axis) were estimated from the fitted dose–response curves based on treatment with five CIGB-325 concentrations determined by cell viability assay (left section). Crystal violet stain wells for each experimental condition in the CPE inhibition assay (right section) scale bars: 3 mm. (**B**) Antiviral effect of positive (IFN-alpha2b, CX-4945) and negative controls (F20.2) was determined by cell viability assay using crystal violet stain. Cells were infected as previously described and incubated with CIGB-325 (30 µM), F20.2 (30 µM), CX-4945 (1.25 µM), and IFN-alpha2b (500 IU). Uninfected cells with the vehicle were set to 100%. C. Progeny titers of the cell culture supernatants collected from CPE inhibition experiments were assessed using plaque assay. Data from (**B**,**C**) are shown as mean ± SD, n = 3. Statistically significant differences between vehicle and drug treatment are represented as ** *p* < 0.01, *** *p* < 0.001 and ns, not significant determined using one-way ANOVA followed Dunnett post-test.

We analyzed the in vitro CIGB-325 effect on BCoV-induced CPE using a cell-based assay. After infection, MDBK cells appeared to be rounded and multinucleated within 48 h, but after 72 h of incubation, cell monolayers were significantly damaged. The incubation of BCoV-Mebus-infected cells with CIGB-325 elicited a very potent inhibition of viral-induced CPE (IC_50_ = 3.5 μM), with just some significant cytotoxicity at higher concentrations (CC_50_ = 150 μM) (Figure 1A) with a Selectivity Index of 42. Specificity of the CIGB-325 antiviral effect was also verified using the F20-2 negative control peptide containing the same cell-penetrating peptide as CIGB-325 fused to peptide lacking CK2 inhibitory effect [14]. IFN alpha-2b was used as an antiviral drug reference. Since previous studies in SARS-CoV-2 infection had shown the antiviral activity of the CK2 inhibitor CX-4945 [12], we also tested its putative anti-BCoV activity. Interestingly, CX-4945 significantly protected the MDBK cells from the BCoV-induced CPE (Figure 1B). After 16 h and 26 h of viral challenge, the treatment with CIGB-325 also showed a clear inhibition of BCoV-induced CPE (Figure S1).

Additionally, we corroborated the CIGB-325 inhibitory effect on BCoV-Mebus infection by measurement of viral load in cell supernatants collected from CPE experiments determined by plaque assay. CIGB-325 displayed dose-dependent inhibition of viral titers, expressed as BCoV-Mebus PFU (Figure 1C).

### 3.2. CIGB-325 Targets BCoV N Protein in MDBK Cells

**Figure 2:**
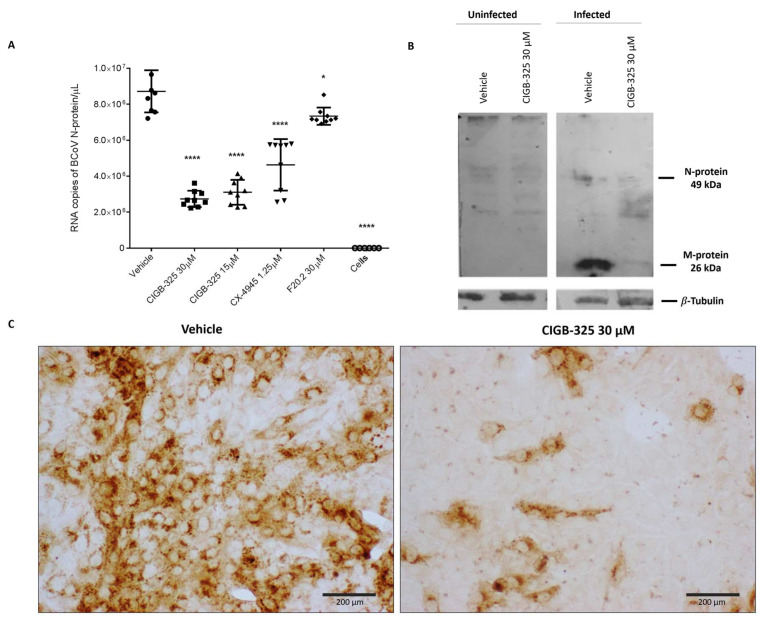
Impact of CIGB-325 on RNA copies of N protein and intracellular protein levels of N and M protein. MDBK cells were treated with the drugs 1 h before the viral challenge. Afterward, cells were infected with BCoV-Mebus (70,000 TCID_50_/well), and the appropriate drug’s concentration was maintained. Effect of CIGB-325 on the RNA copies of N protein and viral M and N proteins levels was investigated using qRT-PCR (**A**) and Western blot (**B**)/ immunocytochemistry (**C**), respectively. (**A**) qRT-PCR reaction was performed with specific oligonucleotides corresponding to N protein. N expression was normalized to *GAPDH* and *HMBS* genes and expressed as RNA copy number obtained after extrapolation in a calibration curve. Values are presented as mean ± SD (n = 9). Statistically significant differences between vehicle and drug treatment are represented as * *p* < 0.05 and **** *p* < 0.0001, determined using one-way ANOVA followed Dunnett post-test. (**B**,**C**). Cell preparations for Western blot and immunocytochemistry were carried out as described in Materials and Methods. A human anti-SARS-CoV-2 polyclonal antibody from one COVID-19 convalescent patient was used for identification of BCoV-Mebus M and N protein. Anti-β-tubulin blot was used as loading control. Viral proteins and β-tubulin protein were blotted in the same gel. Scale bars: 200 µm.

The potential effect of CIGB-325 on RNA expression of the viral N protein was investigated by quantitative real-time PCR (qRT-PCR) in BCoV-Mebus-infected cells. As shown in Figure 2A, CIGB-325 significantly decreased the RNA copy number of N protein after 24 h of incubation in a dose-dependent manner, while F20-2 decreased it slightly. Likewise, CX-4945 treatment affected the nucleocapsid gene expression, which might suggest an involvement of the CK2-mediated phosphorylation into BCoV infection.

We also evaluated CIGB-325’s effect on the M and N viral protein levels. A human anti-SARS-CoV-2 polyclonal antibody from one COVID-19 convalescent patient was used for Western blot experiments. Figure S2 shows that this antibody specifically recognized the M (49 kDa) and N (26 kDa) proteins derived from BCoV. CIGB-325 treatment reduced the viral M-protein levels at 24 h post- BCoV-Mebus infection, although the effect on the N protein levels appeared to be milder (Figure 2B). Immunocytochemistry data revealed a decrease in the intracellular accumulation of viral M and N proteins after treatment with the peptide (Figure 2C). As early as 16 h, CIGB-325 reduced the intracellular levels of these viral proteins (Figure S3).

**Figure 3:**
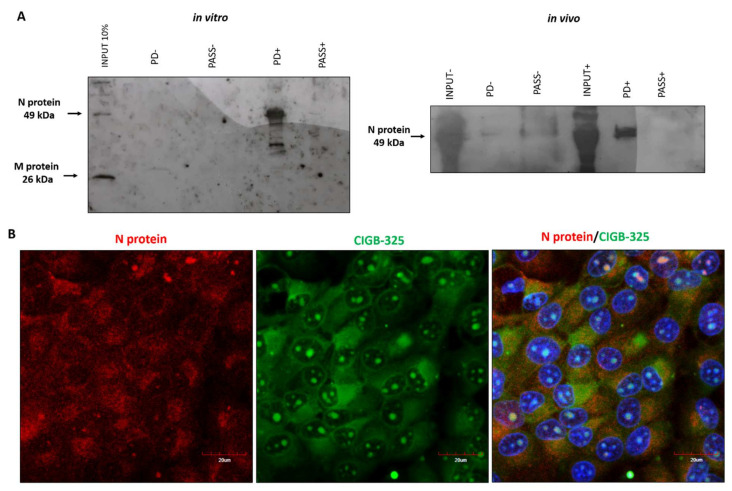
Interaction of CIGB-325 with BCoV-Mebus N protein. (**A**) Western blot analysis of in vitro and in vivo pull-down fractions using CIGB-325 conjugated to biotin as bait to capture interacting proteins. In vitro pull-down was performed with cellular lysates from BCov-Mebus-infected MDBK cells incubated 30 min with biotin-tagged CIGB-325 (100 μM). Subsequently, 30 µL of streptavidin–sepharose was added to each reaction, and the CIGB-325-interacting proteins were eluted, resolved in 12%-SDS-PAGE, and subjected to Western blot to identify the viral N protein. For in vivo pull-down, MDBK cells were treated 30 min with biotin-tagged CIGB-325 (100 μM), subsequently lysed and processed as indicated above. PD: pull-down fractions; PASS: flow-through fractions; NC: negative control (cellular lysate from MDBK cells incubated with the vehicle). (**B**) Representative images obtained by confocal microscopy showing the co-localization of CIGB-325-F with BCoV-Mebus N protein after 30 min of incubation in infected MDBK cells. Red fluorescence: N protein-derived signal; Green: CIGB-325-derived signal; Blue: nuclear DAPI; Orange: merge of Green/Red channels representing co-localization signal. Scale bars: 20 µm.

Considering that CIGB-325 exerts anti-CK2 activity by direct binding to CK2 conserved phosphoacceptor domain on substrates [14], we searched for such aminoacidic motifs on viral proteins to anticipate putative physical interactions. As we confirmed that BCoV-Mebus viral N protein displays such a sequence [35], we conducted in vitro pull-down experiments using biotinylated CIGB-325 to capture interacting proteins from infected-cell lysates. Western blot data from pull-down fractions using the human anti-SARS-CoV-2 polyclonal antibody showed that pull-down fractions, but not the input, displayed a unique band corresponding to BCoV-Mebus N protein (49 kDa) (Figure 3A). In vivo pull-down assays were performed in BCoV-Mebus-infected MDBK cells to confirm the interaction of CIGB-325 with viral N protein on a relevant cellular context. Of note, a clear band corresponding to the N protein (49 kDa) was again observed after Western blot analysis (Figure 3A). To verify the in situ physical proximity between CIGB-325 and N protein at the subcellular compartments, we used confocal microscopy in BCoV-infected cells treated with CIGB-325-F. First of all, we confirmed the suitability of the rabbit polyclonal antibody directed against the SARS-CoV-2 N protein to recognize the BCoV N protein by Western blot and immunocytochemistry (Figure S4). As we anticipated, an orange merged pattern of CIGB-325 with the viral N protein was observed mainly throughout the cytoplasm, with a slighter extent within the nucleus of cells after 30 min of incubation (Figure 3B).

Previous findings have indicated that SARS-CoV-2 N protein directly interacts with protein kinase CK2 [36]. To corroborate such interaction in this in vitro BCoV-Mebus infection model, we conducted immunoprecipitation assays from infected MDBK cell lysates using a commercial anti-CK2 antibody and subsequent Western blot with the rabbit polyclonal anti-SARS-CoV-2 against the BCoV N protein. Data revealed that CK2 also associates physically with the BCoV-Mebus N protein in this cellular context (Figure S5).

### 3.3. CIGB-325 Interactomic Landscape in BCoV-Mebus-Infected MDBK Cells

A full array of viral and host proteins that interact with CIGB-325 in BCoV-Mebus-infected MDBK cells was investigated by in vitro pull-down experiments coupled to LC-MS/MS analysis. We identified 50 host proteins and 2 viral proteins that interact with CIGB-325 in infected MDBK cells. Viral proteins corresponded to N protein and non-structural protein 2a (NSp2a). Members of the family of proteins including actin, tubulin, myosin, heat shock protein 70 (HSP70), heat shock protein 90 (HSP90), and tyrosine 3-monooxygenase/tryptophan 5-monooxygenase activation protein, as well as others shown in Table S2, were identified as host CIGB-325-interacting proteins. In agreement with CIGB-325 interactome from tumor cells [37], nucleolar protein B23/NPM1 was also identified in our study.

**Figure 4:**
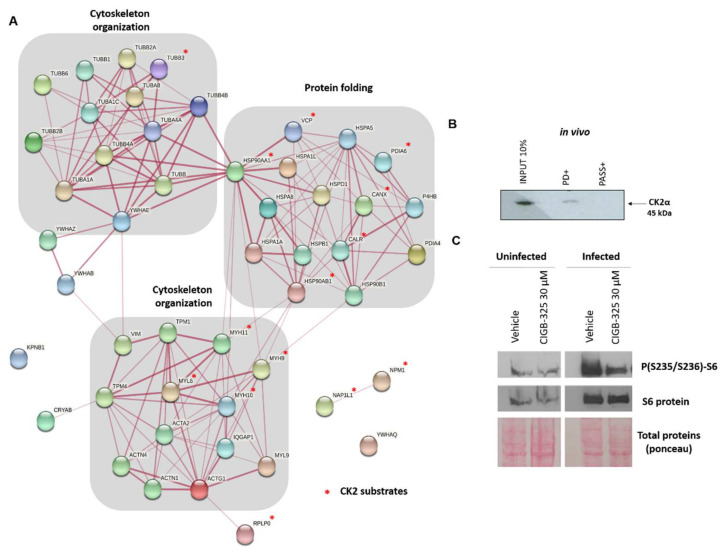
CIGB-325-host interacting proteins in BCoV-infected MDBK cells and effect of the peptide on RPS6 phosphorylation. (**A**) Network was generated using information gathered from the STRING database. Biological processes retrieved from the Gene Ontology database are indicated, and CK2 bona fide substrates are indicated with an asterisk in red. (**B**) In vivo pull-down was performed with biotinylated CIGB-325 as bait to capture CK2 in MDBK-infected cells. Interacting proteins were then resolved by 12% SDS-PAGE, transferred, and each fraction was inspected by Western blot with the antibody against CK2α. (**C**) Effect of CIGB-325 on phosphorylated and total protein levels of RPS6. MDBK cells were infected with BCoV-Mebus (70,000 TCID_50_/well). After 24 h of viral challenge, CIGB-325 was incubated for 45 min, and preparation of cell extracts for Western blot was carried out, as referred to in Material and Methods.

To envisage the putative biological processes that might be perturbed by CK2 targeting, enrichment analysis of the CIGB-325-interacting host proteins was performed. Protein folding and the response to unfolded protein, cytoskeleton organization, and cell cycle were significantly represented in the interactomic profile (Figure S6). We also searched for bona fide CK2 substrates among the CIGB-325-interacting proteins according to the Meggio and Pinna dataset [32], the list of high confidence CK2 substrates reported by Bian et al. [33], and the PhosphoSitePlus database (www.phosphosite.org, accessed on 6 June 2021). CIGB-325 interacted with 15 proteins previously described as CK2 substrates in MDBK cells (Table S2). To better understand the global putative connection among the CIGB-325 interactome in infected MDBK cells, we constructed a protein–protein interaction (PPI) network with the identified proteins, using information annotated in the STRING database (Figure 4A). In the PPI network, we detected two functional physiological complexes corresponding to proteins involved in cytoskeleton organization and protein folding, consistent with the most represented biological processes.

Previous reports from our group have indicated that CIGB-325 also impairs CK2 signaling by direct binding to CK2α catalytic subunit in different cancer cell lines [15,16,38]. We finally determined whether this direct peptide–enzyme interaction could also take place within the context of a viral infection induced by BCoV-Mebus in MDBK cells. Using in vivo pull-down with biotinylated-CIGB-325 followed by Western blot with a commercial anti-CK2α monoclonal antibody, it was possible to demonstrate the presence of CK2α in the pull-down fraction (Figure 4B). To corroborate whether this interaction could impact any of the CK2-mediated signaling pathways, we also evaluated the effect of CIGB-325 on the PI3K/Akt pathway by measurement of the phosphorylation of the downstream RPS6 protein. This protein constitutes a biomarker of the PI3K/Akt pathway activation, which in turn represents one of the CK2-mediated signaling pathways. After 45 min of CIGB-325 treatment, we demonstrated a clear impairment of RPS6 phosphorylation (i.e., at residues S235/236) at 24 h post-BCoV infection in MDBK cells, contrasting with a significant increase in RPS6 phosphorylation upon BCoV infection (Figure 4C).

## 4. Discussion

Coronaviruses represent a threat to animals and humans, causing respiratory or gastrointestinal diseases [2]. BCoV has common characteristics with SARS-CoV-2—both belong to the β-CoV genus and infect respiratory tract and intestine [39,40]. Protein kinase CK2 has been confirmed as a fundamental factor in some steps of the β-CoV life cycle [12,13]. Recently, the interaction of CK2 with the SARS-CoV-2 nucleocapsid protein has been described, which is conserved in MERS-CoV and SARS-CoV [10]. Accordingly, the scientific rationale of using CK2 inhibitor against β-CoV infections could provide clinical benefit for future outbreaks. Therapeutic use of the anti-CK2 peptide CIGB-325 in SARS-CoV-2-infected patients showed clinical benefit; however, whether such clinical effect is due to the direct antiviral effect of CIGB-325 on the SARS-CoV-2-infected lung epithelium remains unclear at present.

Here, using a bovine coronavirus infection model, we explored the potential antiviral effect of CIGB-325 and those molecular events that might support such antiviral effect. CIGB-325 exhibited a clear dose-dependent antiviral activity against BCoV-Mebus in MDBK cells according to two different experimental readouts: inhibition of the cytopathic effect and reduction in the viral titer assessed on plaque assay. Such antiviral effect was achieved at a clinically relevant dose even 16 and 26 h post-viral challenge; further investigation will be required to decipher the steps of the BCoV lifecycle targeted by the peptide [20,41]. Similar to the inhibition of SARS-CoV-2 infection by CX-4945 previously reported on Vero-E6 cells [12], the treatment with this CK2 inhibitor also elicited antiviral effect against BCoV in MDBK cells. We also confirmed that the treatment with both CK2 inhibitors decreased the mRNA levels of BCoV N protein, as determined by qRT-PCR. It remains to be elucidated whether this effect is caused by an inhibition of the viral RNA transcription or it is rather a consequence of targeting other steps of the viral cycle. The treatment with CIGB-325 reduced the levels of M and N protein detected by Western blot and in situ immunocytochemistry, though further experiments to explore the underlying mechanism need to be conducted. Thus, the anti-BCoV activity of two different CK2 inhibitors suggests the relevance of the CK2-mediated phosphorylation during infection by this type of β-CoV.

Recent evidence that has shown a physical interaction and co-localization between the SARS-CoV-2 N protein and protein kinase CK2 [12,36] encouraged us to look for such an interaction in our model. Data from immunoprecipitation experiments clearly corroborated the interaction between CK2 and viral N protein in the context of BCoV-Mebus infection. N protein from both SARS-CoV and SARS-CoV-2 are phosphoprotein with multiple predicted CK2 phosphorylation sites [12,35]; thus, we could speculate that BCoV N protein represents a putative CK2 substrate. In line with that notion, CIGB-325, which binds to the acidic phosphoacceptor domain on the CK2 substrates, interacted with BCoV-Mebus N protein, as evidenced by in vitro and in vivo pull-down experiments. Additional experiments to decipher whether CIGB-325 directly binds to N protein or indirectly through unknown cellular protein complexes are required.

The interaction of CIGB-325 with the viral N protein was confirmed among the CIGB-325-interacting proteins identified by mass spectrometry. Beyond this interaction, we wanted to explore whether other viral and host proteins could be targeted by CIGB-325, thus being relevant for its antiviral activity on BCoV-Mebus infection. We were able to detect the physical interaction of CIGB-325 with the viral protein NSp2a that plays a role in the inhibition of the transcriptional activity of antiviral response elements, including (IFN)-stimulated response element (ISRE), IFN-β promoter, and nuclear factor kappa B (NF-κB) in HCoV-OC43 [42]. Hence, it remains speculative whether part of the CIGB-325 anti-BCoV effect could be explained by upregulation of genes involved in the type I IFN and NF-κB signaling pathways.

Regarding the host identified proteins, those representing functional clusters participating in protein folding, cytoskeleton organization, and cell cycle were the most representative in the constructed protein–protein interaction network as well as in the functional enrichment analysis. All three of these cellular processes are all relevant for the coronavirus lifecycle [12,43,44].

We further interrogated the CIGB-325 interactomic profile for the presence of CK2 substrates and found that 30% (15 proteins) corresponded to previously validated CK2 substrates [32,33]. Three different CK2 substrates targeted by CIGB-325 might be of particular relevance, considering its already known proviral function. That is the case of the B23/NPM1 protein, a major target for CIGB-325 in solid tumor cells [45], which plays an important role as a proviral chaperone in animal and human virus infections [46,47,48]. Analysis of proteomic data from SARS-CoV-2-infected cells has evidenced that B23/NPM1 displays the highest positive correlation with the expression profile of viral proteins [49]. Moreover, the myosin heavy chain 9 (MYH9), which was targeted by CIGB-325, is another bona fide CK2 substrate whose phosphorylation at the S1943 is upregulated during infection of Vero-E6 cells by SARS-CoV-2 [12]. Likewise, the heat shock protein 90 alpha family class B member 1 (HSP90AB1) has been identified as a viral RNA interacting protein significantly upregulated during the SARS-CoV-2 replicative cycle in the RNA-bound proteome in infected lung cells. Consistently, compounds targeting this protein showed a strong inhibition of SARS-CoV-2 protein production [50]. A priori, targeting of the cellular CK2 substrates B23/NPM1, MYH9, and HSP90AB1 along with the viral N protein might support the CIGB-325 antiviral activity against BCoV infection. However, our data do not rule out that other CK2 substrates among CIGB-325-interacting proteins might be also involved in the antiviral activity.

Finally, our in vivo pull-down experiments revealed that CIGB-325 can also interact with CK2 catalytic subunit in MDBK cells, as previously reported in lung cancer and leukemia cells [15,16,38]. CIGB-325 impaired the PI3K/AKT pathway, a CK2-mediated signaling pathway, as evidenced by the phosphorylation of the downstream signaling protein RPS6. Whether this dual anti-CK2 inhibitory mechanism described for CIGB-325 in other cellular contexts could also be running in parallel to mediate antiviral activity remains to be elucidated.

Beyond describing the CIGB-325 antiviral activity against BCoV-Mebus infection and providing clues on the molecular basis supporting such an effect, our data also suggest a clinical benefit of anti-CK2 approaches for treating bovine coronavirus infections.

## 5. Conclusions

In conclusion, we have described for the first time the in vitro antiviral activity of the anti-CK2 peptide CIGB-325 against BCoV-Mebus infection. We found that the CIGB-325 antiviral effect might be supported by targeting host and viral CK2 substrates as well as the CK2 enzyme itself. This pharmacologic effect, together with the favorable risk–benefit balance already observed in clinical trials, reinforces CIGB-325 as a promising drug candidate to be tested in both animal and human β-CoV infections.

## Data Availability

All data are already presented in the manuscript and available on request from the corresponding author.

## Supplementary Materials

The following are available online at https://www.mdpi.com/article/10.3390/v14030552/s1, Figure S1: Antiviral activity of CIGB-325 in late stages of BCoV-Mebus infection, Figure S2: Characterization of polyclonal anti-SARS-CoV-2 antibody for its cross-reactivity with BCoV proteins, Figure S3: Impact of CIGB-325 over the accumulation of N and M protein by immunocytochemistry, Figure S4: Characterization of rabbit polyclonal anti- SARS-CoV-2 N protein antibody for its cross-reactivity with BCoV proteins, Figure S5: Co-immunoprecipitation of BCoV Mebus N protein and CK2, Figure S6: Functional enrichment analysis for CIGB-325-interacting proteins in MDBK infected cells, Table S1: Oligonucleotides sequences, Table S2: CIGB-325-interacting proteins.
