# Supplementary material for: Targeting of Protein Kinase CK2 Elicits Antiviral Activity on Bovine Coronavirus Infection
Source: Viruses. 2022 Mar 7;14(3):552. doi: 10.3390/v14030552 (PMC8949182; doi:10.3390/v14030552)
Supplement: Supplementary file 1 [file viruses-14-00552-s001.zip › Viruses 2022. Supplementary Material.pdf]

Supplementary Material

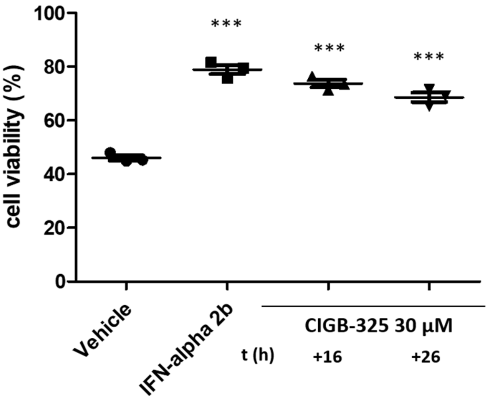

Figure S1. Antiviral activity of CIGB-325 in late stages of BCoV-Mebus infection. MDBK cells were infected with (14 000 TCID50/well), 16 h and 26 h post infection CIGB-325 (30 µM) was added. Afterwards, the cells were incubated for 3 days and the antiviral effect was determined by crystal violet stain. IFN-alpha 2b represent the positive control and was added 1h pre infection as earlier described. Data is shown as mean ± SD, n = 3. Statistically significantly differences between vehicle and drug treatment are represented as ***p* < 0.01 and *** *p* < 0.001 determined using one-way ANOVA followed by Dunnett post-test.

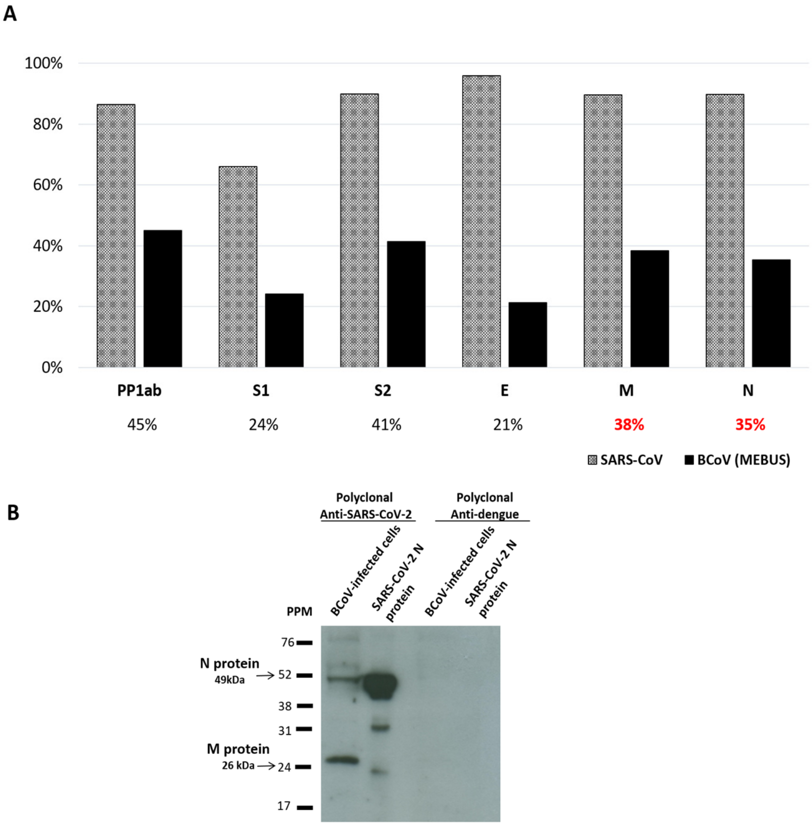

Figure S2. Characterization of polyclonal anti-SARS-CoV-2 antibody for its cross-reactivity with BCoV proteins. A: Similarities scores for SARS-CoV-2 structural proteins compared with BCoV. B: Western blot of BCoV-infected lysates and recombinant SARS-CoV-2 N protein probed with polyclonal anti-SARS-CoV-2 and anti-dengue. MDBK cells were infected with BCoV-Mebus (70 000 TCID50/well). After 24 h post-infection, cell extracts were prepared, resolved in 12%-SDS-PAGE and subjected to western blot to identify the viral proteins. PP1ab:polyprotein 1ab

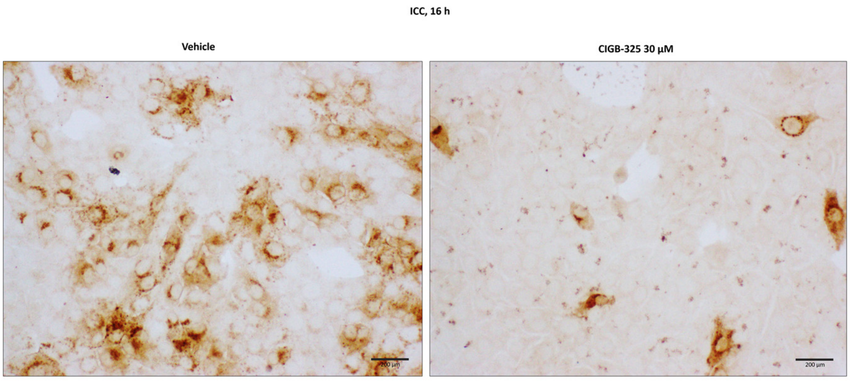

Figure S3. Impact of CIGB-325 over the accumulation of N and M protein by Immunocytochemistry. MDBK cells were treated with the drugs 1 h before the viral challenge. Afterwards, cells were infected with BCoV-Mebus (70 000

TCID50/well) and the appropriate drug's concentration was maintained for 16 h. After the incubation time, cells were fixed and immunostaining using a human anti-SARS-CoV-2 polyclonal antibody, followed by peroxidase conjugated secondary antibody and addition of substrate.

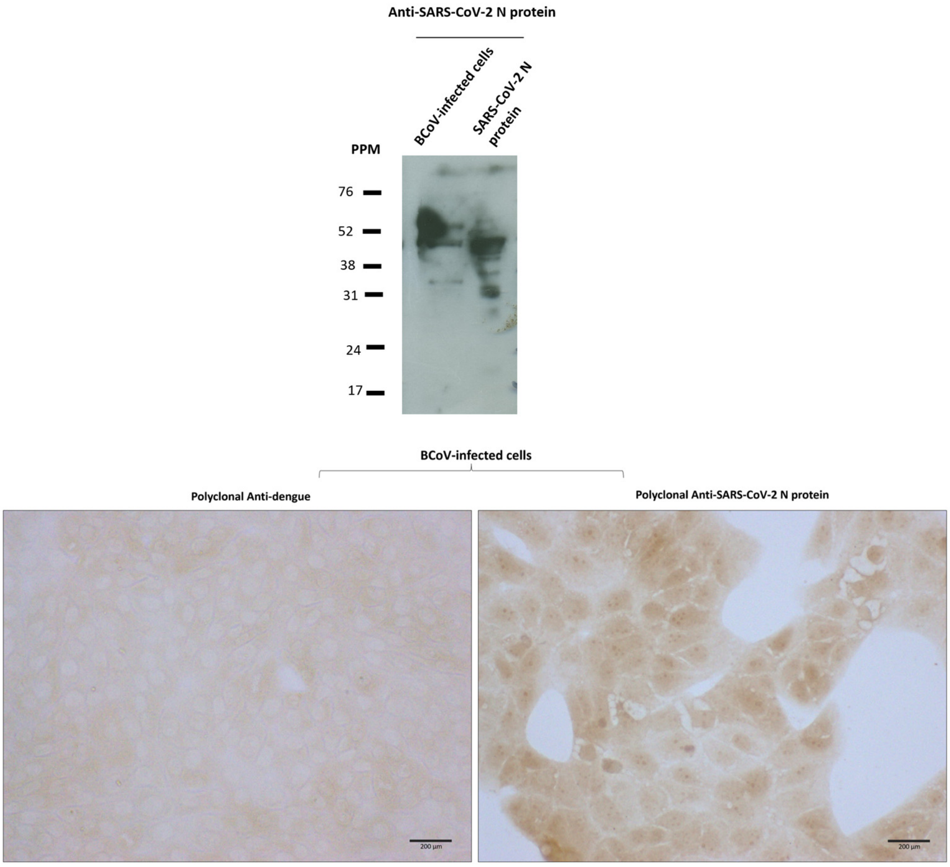

Figure S4. Characterization of rabbit polyclonal anti- SARS-CoV-2 N protein antibody for its cross-reactivity with BCoV proteins. A: Western blot of BCoV-infected lysates and recombinant SARS-CoV-2 N protein probed with polyclonal anti-SARS-CoV-2 N protein antibody. B Immunocytochemistry of BCoV-infected cells using polyclonal anti-SARS-CoV-2 N protein and polyclonal anti-dengue antibodies. After 24 h post-infection, MDBK cells were fixed and stained with the primary antibodies, followed by peroxidase conjugated secondary antibody and addition of substrate.

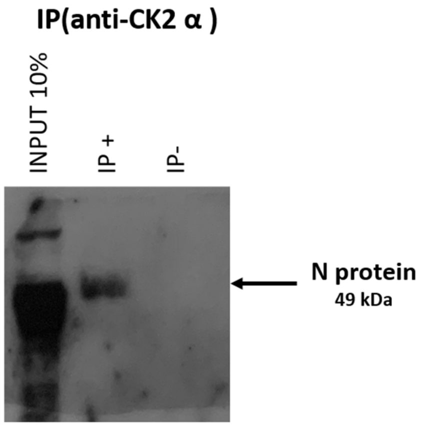

Figure S5. Co-immunoprecipitation of BCoV Mebus N protein and CK2. Cells MDBK were infected 70 000 TCID50. After 48 h post-infection, cell extracts were prepared and immunoprecipitations were performed with anti-CK2α antibody. Cell lysates (lanes marked input) and immunoprecipitates (lanes marked IP) were analyzed on immunoblots with anti- SARS-CoV-2 N protein polyclonal antibody. The position of the N protein is indicated by an arrow. Nonspecific bands with anti-N protein antibody are shown.

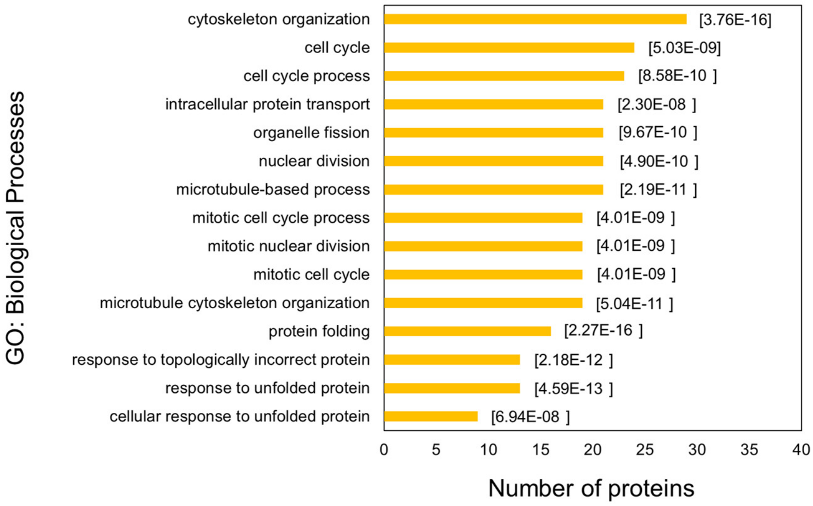

Figure S6. Functional enrichment analysis for CIGB-300 interacting proteins in MDBK infected cells. Biological processes significantly represented in CIGB-300 interactome were identified using annotations from Gene Ontology database. Analysis was performed with ToppFun web-based tool (www.toppgene.cchmc.org/enrichment.jsp/). The *p*-value of each annotation is placed in square brackets.

Table S1. Oligonucleotides sequence

**Oligonucleotides**	**Sequences 5'-3'**	**Number of bases**
bovine GAPDH	AGATGGTGAAGGTCGGAGTG	20
bovine GAPDH	TGGAAGATGGTGATGGCCTT	20
bovine HMBS	TGTTGCACGATCCTGAGACT	20
bovine HMBS	TTCATGCTGGACAGGGACAT	20
bovine N protein	ATTTGCAGAGGGACAAGGTG	20
bovine N protein	GCGGTCCTGTTCCAAGATAG	20
