# Supplementary figures and images for: Targeting of Protein Kinase CK2 Elicits Antiviral Activity on Bovine Coronavirus Infection
Source: Viruses. 2022 Mar 7;14(3):552. doi: 10.3390/v14030552 (PMC8949182; doi:10.3390/v14030552)

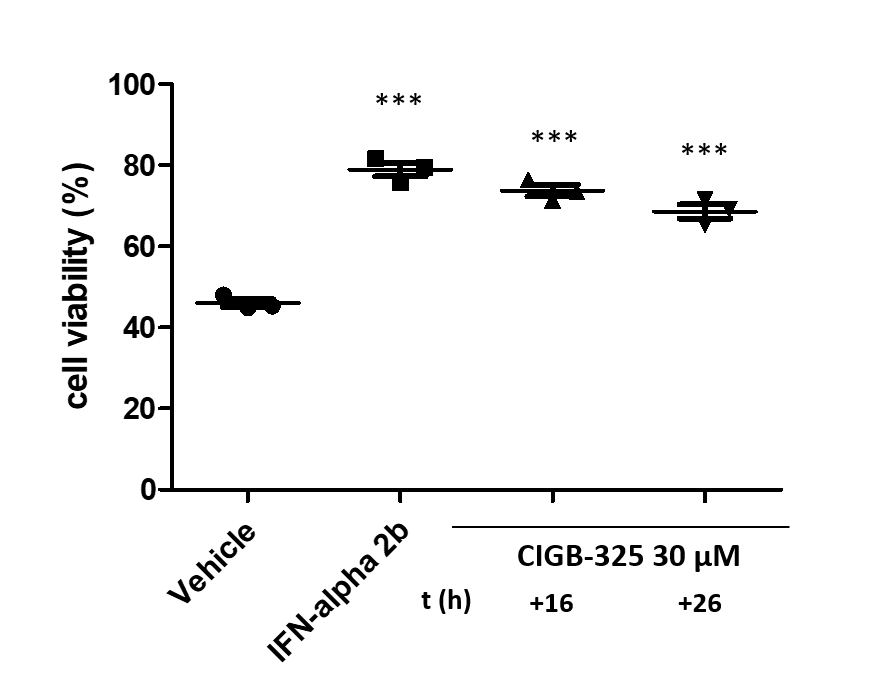

Supplement: Supplementary file 1 [file viruses-14-00552-s001.zip › FigS1.png]

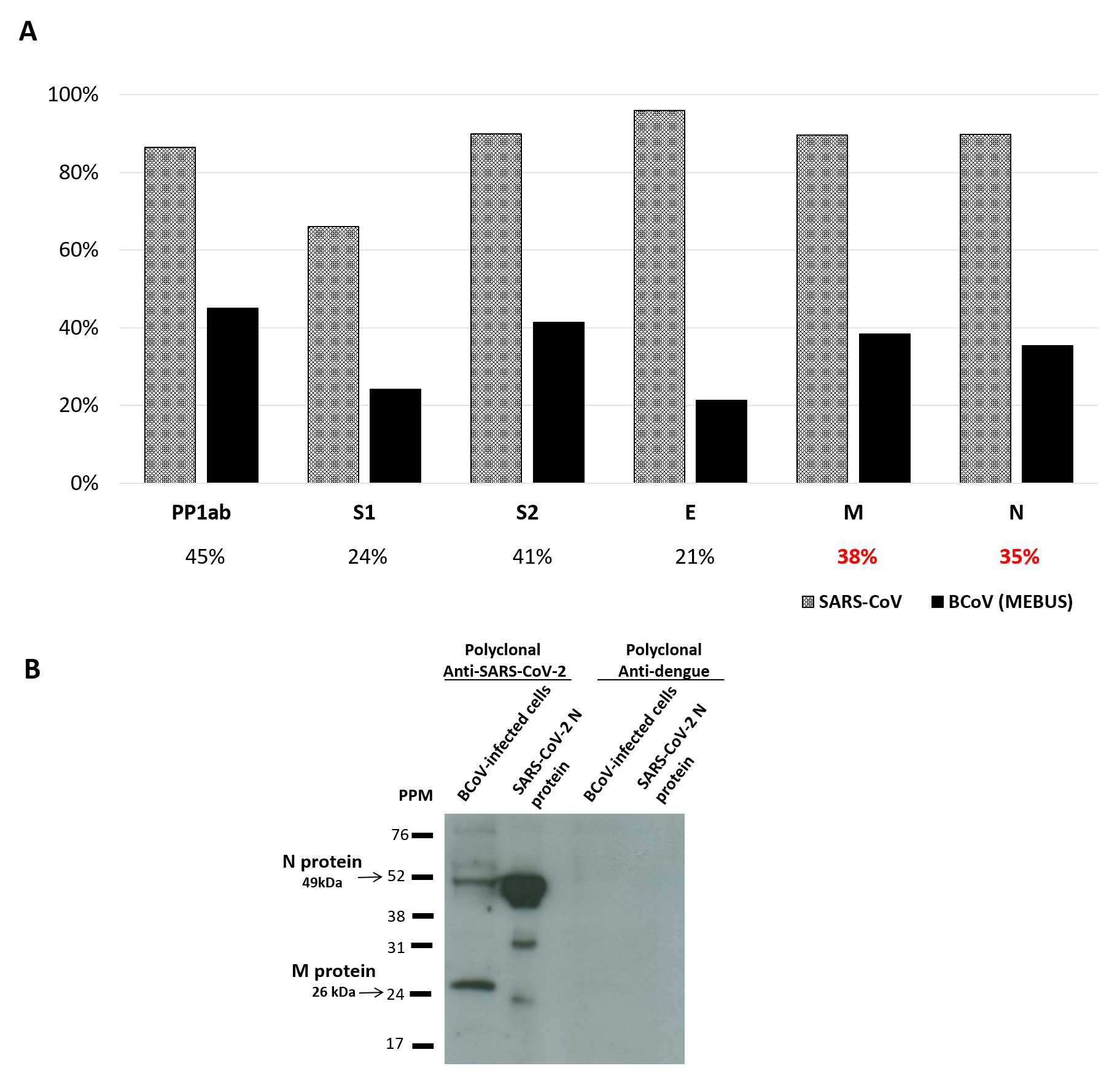

Supplement: Supplementary file 1 [file viruses-14-00552-s001.zip › FigS2.png]

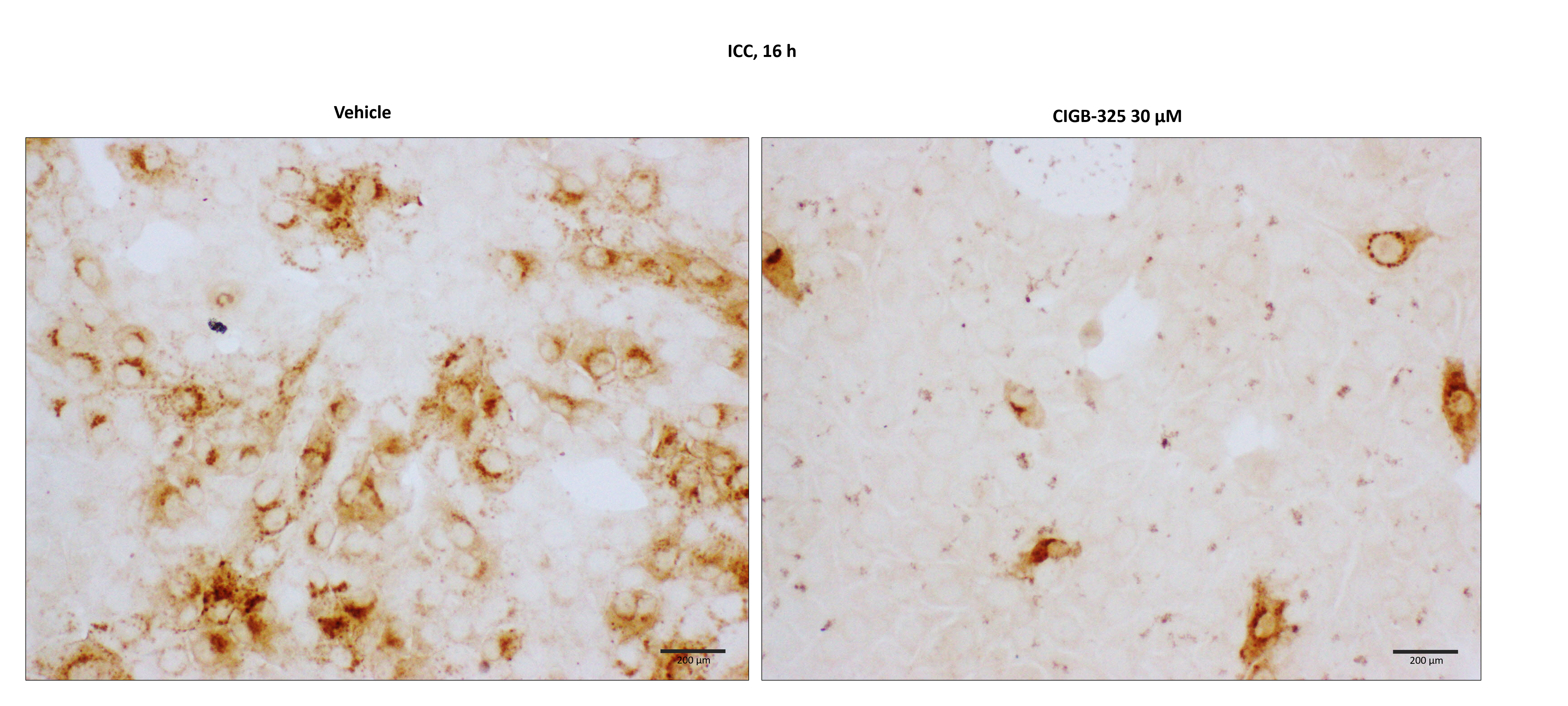

Supplement: Supplementary file 1 [file viruses-14-00552-s001.zip › FigS3.png]

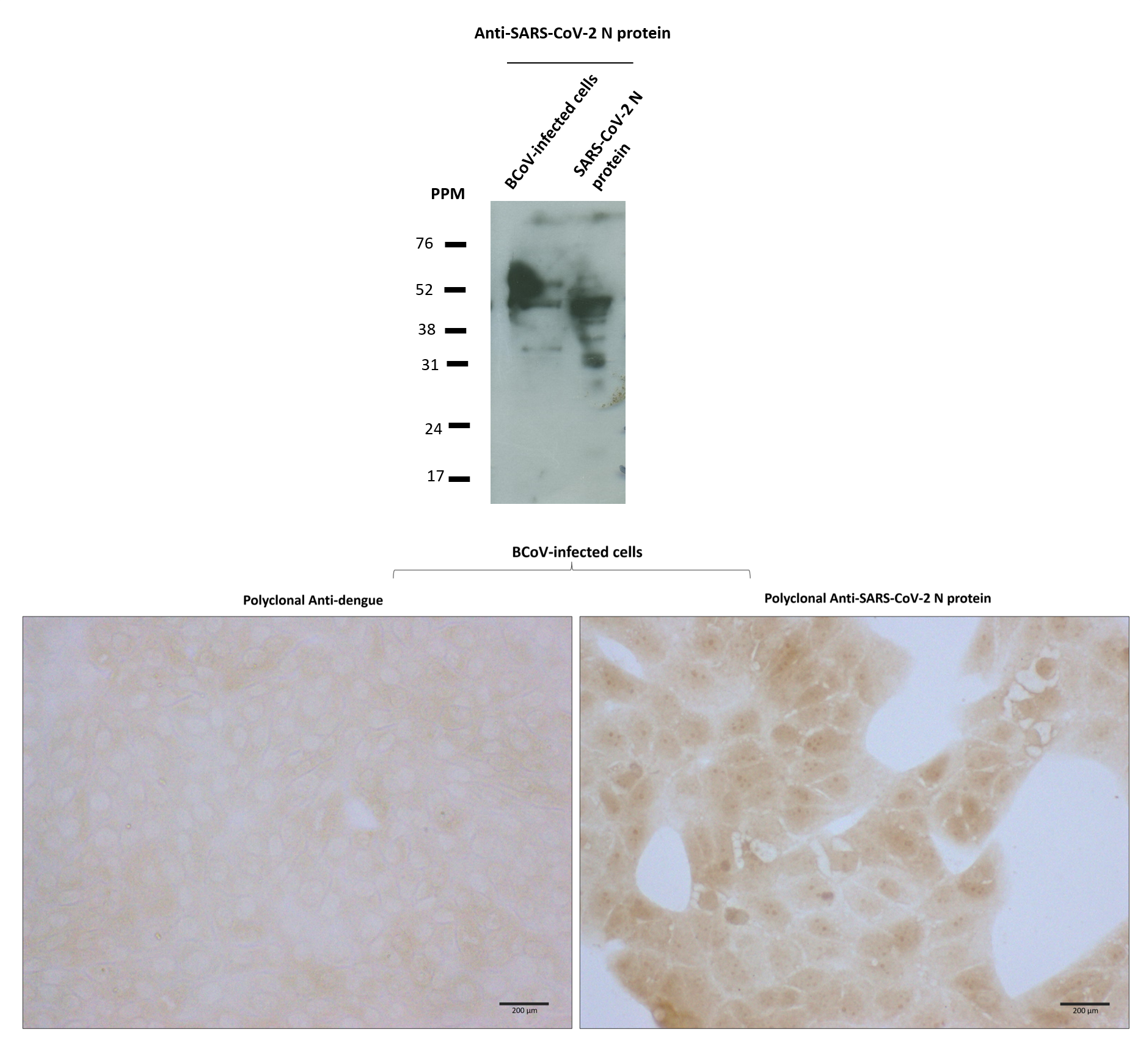

Supplement: Supplementary file 1 [file viruses-14-00552-s001.zip › FigS4.png]

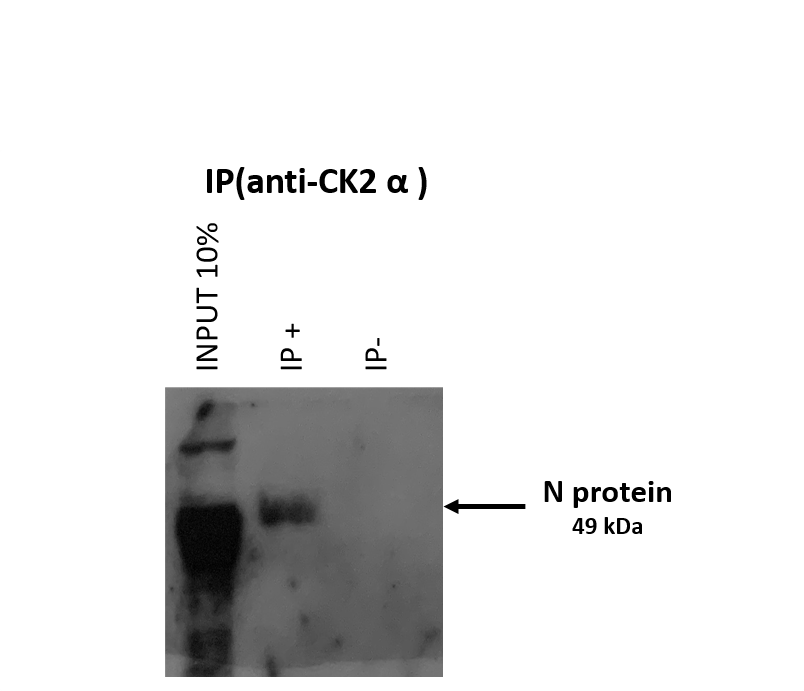

Supplement: Supplementary file 1 [file viruses-14-00552-s001.zip › FigS5.png]

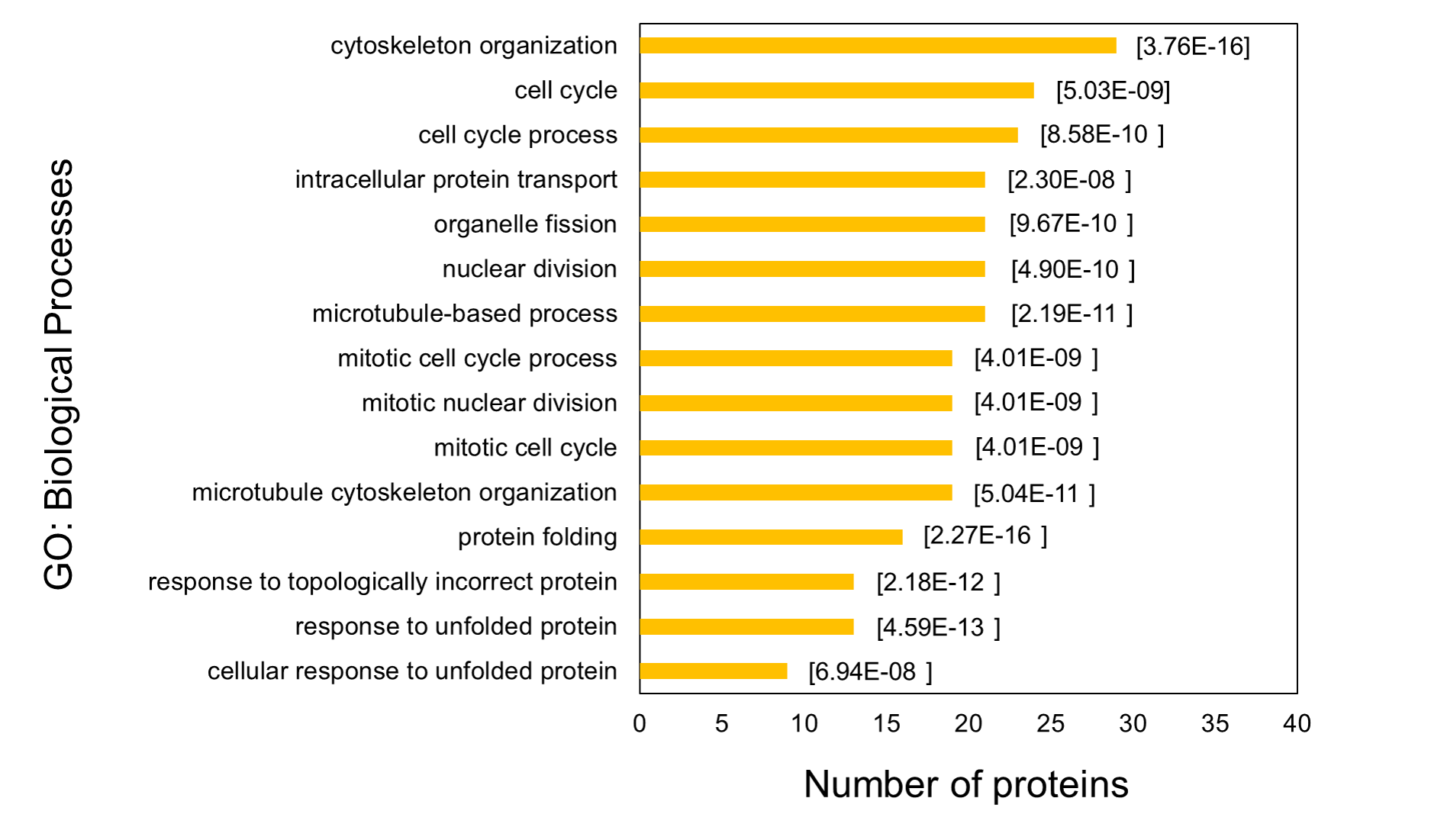

Supplement: Supplementary file 1 [file viruses-14-00552-s001.zip › FigS6.png]
